# Development and optimization of quantitative PCR for the diagnosis of invasive aspergillosis with bronchoalveolar lavage fluid

**DOI:** 10.1186/1471-2334-8-73

**Published:** 2008-05-29

**Authors:** Prasanna D Khot, Daisy L Ko, Robert C Hackman, David N Fredricks

**Affiliations:** 1 Fred Hutchinson Cancer Research Center, Seattle, WA, USA; 2 University of Washington, Seattle, WA, USA

## Abstract

**Background:**

The diagnosis of invasive pulmonary aspergillosis (IPA) remains challenging. Culture and histopathological examination of bronchoalveolar lavage (BAL) fluid are useful but have suboptimal sensitivity and in the case of culture may require several days for fungal growth to be evident. Detection of *Aspergillus *DNA in BAL fluid by quantitative PCR (qPCR) offers the potential for earlier diagnosis and higher sensitivity. It is important to adopt quality control measures in PCR assays to address false positives and negatives which can hinder accurate evaluation of diagnostic performance.

**Methods:**

BAL fluid from 94 episodes of pneumonia in 81 patients was analyzed. Thirteen episodes were categorized as proven or probable IPA using Mycoses Study Group criteria. The pellet and the supernatant fractions of the BAL were separately assayed. A successful extraction was confirmed with a human 18S rRNA gene qPCR. Inhibition in each qPCR was measured using an exogenous DNA based internal amplification control (IAC). The presence of DNA from pathogens in the *Aspergillus *genus was detected using qPCR targeting fungal 18S rRNA gene.

**Results:**

Human 18S rRNA gene qPCR confirmed successful DNA extraction of all samples. IAC detected some degree of initial inhibition in 11 samples. When culture was used to diagnose IPA, the sensitivity and specificity were 84.5% and 100% respectively. Receiver-operating characteristic analysis of qPCR showed that a cutoff of 13 fg of *Aspergillus *genomic DNA generated a sensitivity, specificity, positive and negative predictive value of 77%, 88%, 50%, 96% respectively. BAL pellet and supernatant analyzed together resulted in sensitivity and specificity similar to BAL pellet alone. Some patients did not meet standard criteria for IPA, but had consistently high levels of *Aspergillus *DNA in BAL fluid by qPCR.

**Conclusion:**

The *Aspergillus *qPCR assay detected *Aspergillus *DNA in 76.9% of subjects with proven or probable IPA when the concentrated BAL fluid pellet fraction was used for diagnosis. There was no benefit from analyzing the BAL supernatant fraction. Use of both extraction and amplification controls provided optimal quality control for interpreting qPCR results and therefore may increase our understanding of the true potential of qPCR for the diagnosis of IPA.

## Background

Invasive pulmonary aspergillosis (IPA) is a common infection in patients with hematological malignancies and those undergoing hematopoietic cell transplantation [1]. Despite the availability of several active antifungal agents, IPA continues to have a high mortality rate [2]. The diagnosis of IPA remains a challenge [3,4]. Most symptoms are non-specific, such as fever, cough, or chest pain, and many patients have no symptoms at all. Although some radiographic findings in the lungs can suggest aspergillosis, such as the presence of a halo sign (ground glass opacity surrounding a nodule) or cavitating nodules, these findings can also be found in subjects with pulmonary zygomycosis or other infections and are thus again not necessarily specific [5]. The failure to make an accurate diagnosis frequently results in the use of empirical antifungal therapy in the suitable immunocompromised host.

Bronchoalveolar lavage (BAL) fluid is routinely used to assess for the presence of fungi at the site of pulmonary infection. Conventional microbiological techniques like culture and histology of BAL fluid are most commonly used for the diagnosis of IPA, but have suboptimal sensitivity, and in the case of culture may take several days [6-8]. Detection of the fungal cell wall constituents like galactomannan (in serum and BAL fluid) and beta-glucan (in serum) are promising diagnostic alternatives to facilitate the diagnosis of invasive fungal infection, but false positive and false negative results remain problematic with both assays [9-11]. Molecular diagnostic techniques such as nucleic acid detection by PCR are emerging as potentially more sensitive and rapid alternatives to conventional techniques for the diagnosis of IPA [12-19]. Our study focuses on developing a quantitative PCR (qPCR) platform to detect *Aspergillus *DNA in BAL as an indicator of IPA.

Quantitative PCR has several advantages when used for the detection of *Aspergillus spp*. First, qPCR is highly sensitive with the potential to detect a few gene copies per reaction, or less than a single genome for multicopy genes such as the rRNA gene. Second, by taking advantage of both conserved and variable regions of genes, primers and probes can be made that are specific for a given genus, species or strain of microbe. Third, qPCR can measure the amount of microbial DNA in a clinical sample, which may be useful for assessing the burden of infection and in distinguishing between colonization and infection. Fourth, multiplexed qPCR reactions can reduce the necessity of running independent qPCRs allowing for the detection of multiple targets or for inclusion of amplification controls in a single reaction. Fifth, qPCR assays can be completed in a few hours, resulting in a rapid turn around time for reporting results. However, to develop an optimal qPCR assay for diagnosis, several challenges and shortcomings should be addressed to avoid false positive and false negative results [20-22]. False negatives can occur due to suboptimal DNA extraction (i.e. low recovery of DNA and/or the presence of PCR inhibitors), large quantities of human genomic DNA competing with the microbial target for amplification, and suboptimal analytical sensitivity of the qPCR reaction itself (high detection threshold). False positives can occur due to introduction of contamination during sample collection, DNA extraction, and PCR set-up, resulting from the presence of fungi in the environment or fungal PCR product carry-over. In addition, false positives can occur in the setting of suboptimal analytical specificity in the qPCR, resulting from cross-reactivity of the target qPCR assay with other (non-target) fungi or DNA. Optimal qPCR assays should incorporate controls to assess for these factors contributing to false positive and false negative results, but most studies published to date have not. A major hurdle to the wider adoption of PCR for the diagnosis of invasive fungal infections is the lack of standardization among assays. It is important that investigators in the field adopt standards for ruling out false positive and false negative results, as this will allow for more accurate comparisons among different assay platforms.

Many studies have used BAL fluid for the diagnosis of fungal pneumonia using PCR, but the best fraction of BAL fluid to use for this purpose has not been defined. It is unclear if the majority of *Aspergillus *DNA in BAL fluid is contained in intact cells or is cell-free DNA that results from lysis of fungi in vivo. We hypothesized that if most of the *Aspergillus *DNA is present in intact cells, then detection can be maximized by concentrating the cellular fraction of the BAL into a pellet by centrifugation before subjecting this smaller volume to DNA extraction. If cell-free *Aspergillus *DNA is the dominant form in BAL fluid, then qPCR should detect equal or greater amounts of DNA in this larger supernatant fraction.

To optimize our qPCR assay platform for the diagnosis of IPA, we developed a panel of qPCR assays, including amplification and extraction controls, and modified a DNA extraction technique to increase yields of fungal DNA from BAL fluid. This optimized assay was tested on sequentially obtained BAL samples collected from patients with hematological malignancies or undergoing hematopoietic cell transplantation at the Seattle Cancer Care Alliance who developed pneumonia or pulmonary nodules. In an initial analysis, both the pellet and supernatant fractions of the BAL were assayed to determine the optimal fraction for detecting *Aspergillus *DNA. The performance of our qPCR assay was compared with the conventional microbiological techniques of culture and histology.

## Methods

### Study population and design

**Figure 1:**
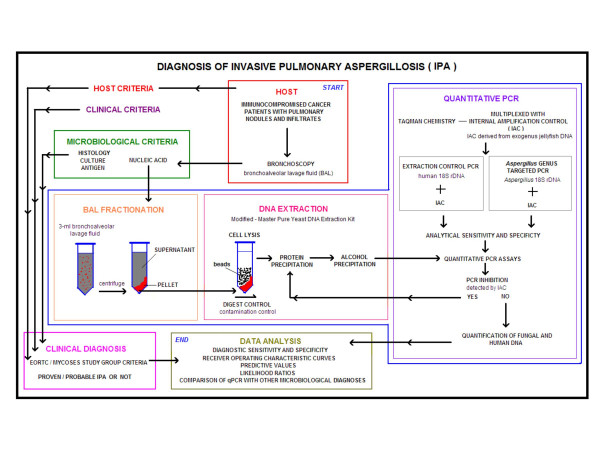
**Diagnostic approach**. Flowchart depicting the algorithm used for the diagnosis of IPA using qPCR.

Patients with hematological malignancies or undergoing hematopoietic cell transplantation at the Seattle Cancer Care Alliance who developed pneumonia or pulmonary nodules underwent bronchoscopy with BAL. BAL fluid remaining after conventional microbiological and cytologic evaluations was processed as noted in the next sub-section. This was a retrospective study analyzing BAL fluid samples obtained from April 2002 to July 2003, and was approved by the Institutional Review Board at the Fred Hutchinson Cancer Research Center. This study involved 81 patients, 94 episodes of pneumonia, and 144 BAL samples. Note that multiple lobes were lavaged at the time of bronchoscopy in most subjects, resulting in an average of more than one BAL sample per episode. Analysis was done on an episode basis, with an episode defined as a single radiographically and temporally related pneumonia. If a subject had resolution of pulmonary infiltrates with appearance of a new infiltrate at a later time, this was considered a separate episode. Figure 1 depicts the algorithm used for the diagnosis of IPA using qPCR. Patients with proven or probable IPA were diagnosed using European Organization for Research and Treatment of Cancer/Mycoses Study Group (EORTC/MSG) criteria [23]. Designation of clinical status was performed by an investigator who was blinded to qPCR results, with host factors, clinical criteria, and microbiological criteria abstracted from the medical record and entered into a relational database.

### Processing of BAL fluid

The starting volume of BAL fluid was in the range of 2 to 5 ml. BAL fluid was centrifuged at 3200 rcf for 15 min at 4°C. The pellet was resuspended in a small volume of supernatant, with the final pellet fraction having a volume of 100 to 400 μl, depending on the degree of cellularity. The pellet and the remaining supernatant fraction were frozen in separate tubes at -80°C until DNA extraction.

### DNA extraction from BAL fractions

DNA extraction of clinical samples and PCR set up was performed in a laminar flow hood within a laboratory that was exclusively used for pre-PCR processing. An optimized version of the MasterPure™ Yeast DNA Purification Kit (Epicentre^® ^Biotechnologies, Madison, WI) was used for BAL DNA extraction. The 100% isopropanol, 70% ethanol and DNA grade water used for extraction were filtered in an Amicon Ultra-15 centrifugal filter unit with a molecular weight cut-off (MWCO) of 30 kDa (Millipore Corporation, Billerica, MA). Yeast Cell Lysis™ solution and MPC Protein Precipitation Reagent™ were UV irradiated at 240 mJ/cm^2 ^with samples approximately 15 centimetres from the bulbs (Spectrolinker™, Westbury, NY). The silicon carbide sharps were washed 10 times in DNA free water and baked at 180°C for 48 h. DNA-free microcentrifuge tubes were used with DNA extraction (Eppendorf Biopur tubes, Eppendorf AG, Hamburg, Germany). Sham digest controls consisting of DNA free water were processed with every extraction run serving as negative controls to monitor for fungal contamination.

DNA was independently extracted from the pellet and supernatant fractions of the BAL; no whole BAL was processed. In the case of the supernatant fraction, extraction started with 0.5 ml of the supernatant from the protein precipitation step onwards. For the pellet fraction, an additional bead beating step was included. Two milliliter sterile screw-cap tubes were loaded with silicon carbide sharps of sizes 0.1 mm and 1 mm (BioSpec Products, Inc., Bartlesville, OK) at a 1:1 ratio up to a volume equivalent to 250 μl. Yeast Cell Lysis™ solution at a volume of 550 μl and BAL pellet at 100 – 400 μl, or 200 μl of water as digest control, were added to the tube. The contents of the tube were homogenized in a FastPrep^®^-24 System (MP Biomedicals, Solon, OH) at 5 m/s for 60 s. Each tube was incubated at 65°C for 45 min then kept on ice for 5 min. MPC Protein Precipitation Reagent™ was added at a volume of 325 μl for pellet and 450 μl for supernatant processing. The tubes were vortexed for 10 s and centrifuged at 11,000 rcf for 10 min. The resulting supernatant was transferred to a new micro-centrifuge tube containing an equal volume of 100% isopropanol pre-cooled to -20°C. The contents of the tube were mixed thoroughly by inversion and incubated at -20°C for 1 hour. Precipitated DNA was pelleted by centrifugation at 11,000 rcf for 10 min. This supernatant was removed and discarded. The pellet containing DNA was resuspended in 0.5 ml of pre-cooled (-20°C) 70% ethanol and vortexed. The tube was then centrifuged at 11,000 rcf for 5 min. This supernatant was removed to a level just short of disturbing the pellet. The remaining volume of ethanol was allowed to evaporate by air drying for 5 min within the laminar flow hood. The pellet was resuspended in 100 μl of 0.1% Triton-X pre-warmed to 65°C then incubated at room temperature for one minute with periodic gentle vortexing. The DNA was either used immediately for qPCR, stored at -20°C overnight or at -80°C for longer periods. If PCR inhibition was detected in the extracted samples, they were reprocessed from the protein precipitation step onwards (see Figure 1).

### Preparation of fungal genomic DNA

Genomic DNA from fungi was extracted with an optimized MasterPure™ Yeast DNA Purification Kit (Epicentre^® ^Biotechnologies, Madison, WI) in order to assess assay analytical sensitivity and specificity. Fungi were transferred into micro-centrifuge tubes from liquid media and centrifuged. Cell pellets were washed with 1 ml 1× PBS and centrifuged at 10,000 rcf for 3 min. The supernatant was discarded and cells resuspended in 500 μl Yeast Cell Lysis™ solution. The tube was vortexed at top speed for 10 s. The tube was incubated at 65°C for 1 h and then kept on ice for 5 min. For filamentous fungi, the pellet was ground with a micropestle at the start and during the 65°C incubation. Protein Precipitation Reagent™ was added at a volume of 400 μl to the tube and vortexed for 10 s. The tube was centrifuged to pellet cellular debris at 11,000 rcf for 10 min. The supernatant was transferred to a new micro-centrifuge tube containing an equal volume of 100% isopropanol pre-cooled to -20°C. The contents of the tube were thoroughly mixed by inversion and incubated at -20°C for 1 hr. Precipitated DNA was pelleted by centrifugation at 11,000 rcf for 10 min. The supernatant was removed and discarded. The pellet containing DNA was resuspended in pre-cooled (-20°C) 1 ml of 70% ethanol and vortexed at maximum speed for 10 s. The tube was then centrifuged at 11,000 rcf for 5 min. This supernatant was removed to a level just short of disturbing the pellet. The remaining volume was allowed to evaporate by air drying for 5 min. The pellet was resuspended in 100 μl of 0.1% Triton-X pre-warmed to 65°C and incubated at room temperature for 1 min with periodic gentle vortexing. The total nucleic acid in the extract was quantified using a UV spectrophotometer. For every 149 μg of total nucleic acid in the extract, 10 U of RiboShredder™ RNase Blend (Epicentre^® ^Biotechnologies, Madison, WI) was used to remove RNA. RNA removal was confirmed by visualizing the pre- and post-treatment extract on a 1.5% agarose gel. DNA was quantified using a Qubit™ instrument and Quant-iT™ dsDNA HS Assay Kit (Invitrogen Corporation, Carlsbad, CA).

### Quantitative PCR assays

Quantitative PCR assays in this study were based on Taqman™ chemistry and an Applied Biosystems 7500™ real-time instrument was used for detection. To prevent contamination, each PCR master mix without additional water component was filtered through a Microcon YM-100 centrifugal filter unit with a MWCO of 100 kDa (Millipore Corporation, Billerica, MA) at 650 rcf for 25 min and 1500 rcf for an additional 5 min before use. The additional water was independently filtered with an Amicon Ultra-15 centrifugal filter unit with a MWCO of 30 kDa using. DNA-free microcentrifuge tubes were used with the PCR set up (Eppendorf Biopur tubes, Eppendorf AG, Hamburg, Germany). No-template controls were run with each qPCR assay to monitor contamination. Each extracted BAL sample was run in duplicate reactions. Samples were interpreted as positive if both duplicates showed an increase in normalized relative florescence above the background and the multicomponent view demonstrated an increase in absolute florescence (as estimated by the 7500 System SDS software, Applied Biosystems).

#### (i) Internal amplification control (IAC) qPCR

The IAC qPCR was developed based on a 105 base template derived from the jellyfish aequorin gene which has a sequence of 5'- GCCTGGTGCAAAAATTGCTTATCAAATTGAACGGTCAATTGGAAGTGGCGGAAGAACAGCTATTGCAAACGC

CATCGCACAATACCATAAACACACTTGTCTTAG-3' [24]. The amplicon was detected with a forward primer 5'-GCC TGG TGC AAA AAT TGC TTA TC-3', reverse primer 5'- CTA AGA CAA GTG TGT TTA TGG TAT TG -3' and probe labelled with fluorescein (Quasar670) and quenched with BHQ2: 5'-Quasar670 CTT CCG CCA CTT CCA ATT GAC CGT TCA BHQ2-3' (Biosearch Technologies, Novato, CA). The IAC was multiplexed with the *Aspergillus *targeted 18S qPCR and the human targeted 18S extraction control qPCR to monitor inhibition in every qPCR reaction. If inhibition as assessed by > 2 cycle delay in the IAC threshold cycle was detected, DNA was re-purified and assayed again.

#### (ii) Extraction control qPCR

Successful DNA extraction was confirmed in all samples with a qPCR targeting the human 18S rRNA gene with forward primer 5'- CTC TTA GCT GAG TGT CCC GC -3', reverse primer 5'- CTT AAT CAT GGC CTC AGT TCC GA -3', and probe labelled with fluorescein (FAM) and quenched with TAMRA: 5'-FAM CCG AGC CGC CTG GAT ACC GCA GCT A TAMRA-3'. Each 50-μl PCR mixture contained 1× TaqMan^® ^Buffer A, 6 mM of MgCl_2_, 1 mM of GeneAmp^® ^dNTP Blend (12.5 mM with dUTP), 2.2 U of AmpliTaq Gold^® ^DNA Polymerase, 0.05 U AmpErase^® ^Uracil N-glycosylase (all from Applied Biosystems, Foster City, CA), 0.8 μM each of forward and reverse human targeted primers, 180 nM of human targeted probe, 0.24 μM each of forward and reverse of IAC primers, 180 nM of IAC probe, 0.002% of Triton-X 100, 10^5 ^copies of IAC template and 5 μl of DNA. The PCR cycling conditions consisted of an Uracil N-glycosylase activation at 50°C for 2 min, pre-melt at 95°C for 10 min and then 38 cycles of 95°C for 15 s (melt) and 65°C for 65 s (annealing and extension). A standard curve for quantifying human DNA was generated using human genomic DNA (Roche Applied Sciences, Indianapolis, IN) with dilutions ranging from 10,000 to 1 pg.

#### (iii) *Aspergillus *targeted 18S qPCR

The *Aspergillus *targeted qPCR amplified a 114 bp segment of the *Aspergillus *18S rRNA gene with forward primer 5'- GAT AAC GAA CGA GAC CTC GG -3', reverse primer 5'- AGA CCT GTT ATT GCC GCG C -3' and probe 5'-FAM CTT AAA TAG CCC GGT CCG C BHQ-3' with minor groove binding modification. Each 50-μl PCR mixture contained 1× TaqMan^® ^Buffer A, 6 mM of MgCl_2_, 1 mM of GeneAmp^® ^dNTP Blend (12.5 mM with dUTP), 2.2 U of AmpliTaq Gold^® ^DNA Polymerase, 0.05 U AmpErase^® ^Uracil N-glycosylase (all from Applied Biosystems, Foster City, CA), 0.8 μM each of forward and reverse *Aspergillus *targeted primers, 200 nM of *Aspergillus *targeted probe, 0.4 μM each of forward and reverse of IAC primers, 190 nM of IAC probe, 0.002% of Triton-X 100, 10^5 ^copies of IAC template and 5 μl of DNA. The PCR cycling conditions consisted of an Uracil N-glycosylase activation at 50°C for 2 min, pre-melt at 95°C for 10 min and then 45 cycles of 95°C for 15 s (melt) and 65°C for 65 s (annealing and extension). A standard curve for quantifying *Aspergillus *DNA was generated using *Aspergillus fumigatus *genomic DNA (ATCC # MYA-1163) dilutions ranging from 1000 pg to 30 fg. All positive *Aspergillus *qPCRs for the first 48 episodes were subjected to sequencing using Big Dye terminators and an Applied Biosystems capillary sequencer to confirm identity with the expected target.

### Analytical specificity testing

The analytical specificity of the *Aspergillus *qPCR was assessed by testing 1000 pg of genomic DNA from 29 different fungal species spanning 15 genera grown in culture. The following clinically or phylogenetically relevant fungal pathogens were chosen: *Aspergillus fumigatus *(ATCC # MYA-1163), *Aspergillus oryzae *(ATCC # 20719), *Aspergillus ustus *(ATCC # 20063), *Aspergillus candidus *(ATCC # 20022), *Aspergillus terreus *(ATCC # 10070), *Aspergillus flavus *(ATCC # MYA-3631), *Candida albicans *(ATCC # 90028), *Candida glabrata *(ATCC # 90876), *Candida kefyr *(ATCC # 28838), *Candida guilliermondii *(ATCC # 90877), *Candida lusitaniae *(ATCC # 42720), *Candida dubliniensis *(ATCC # MYA-580), *Scedosporium apiospermum *(ATCC # 28206), *Scedosporium prolificans *(ATCC # 90468), *Paecilomyces variotti *(ATCC # 10865), *Penicillium chrysogenum *(ATCC # 10108), *Rhizopus oryzae *(ATCC # 10260), *Rhodotorula glutinis *(ATCC # 16726), *Absidia corymbifera *(ATCC # 14058), *Fusarium solani *(ATCC # 56480), *Mucor racemosus *(ATCC # 42647), *Rhizomucor miehei *(ATCC # 46345), *Cunninghamella bertholletiae *(ATCC # 42155), *Trichosporon cutaneum *(ATCC # 38300), *Candida parapsilosis *(clinical isolate), *Candida tropicalis *(clinical isolate), *Candida krusei *(clinical isolate), *Saccharomyces cerevisiae *(Novagen, Madison, WI), and *Cryptococcus neoformans *(ATCC # 28958D-5). Cross-reactivity with 1 μg of human genomic DNA was also assessed.

### Data analysis

Quantitative PCR results were compared with clinical diagnoses based on the EORTC/MSG criteria. Sensitivity, specificity and positive and negative likelihood ratios with their associated 95% confidence intervals were calculated. The negative and positive predictive values (NPV and PPV) were also calculated for these sequentially obtained samples. These diagnostic parameters were also calculated for culture, histology and both culture and histology combined. A receiver-operating characteristic (ROC) analysis was done using a computer program written with MathWorks MATLAB^® ^software to assess how changing qPCR detection threshold affects sensitivity and 1-specificity.

## Results

### Demographic characteristics of the patient population

Of the 81 subjects with pneumonia or pulmonary nodules studied, 60 (74.1%) underwent hematopoietic cell transplantation and the remainder of the subjects had a diagnosis of leukaemia, lymphoma or another neoplastic condition (see Table 1). Accordingly, the study population represents a group of patients at very high risk for invasive aspergillosis based on risk factors such as underlying malignancy, neutropenia and use of steroids.

**Table 1:** Demographic characteristics in 81 subjects.

**Characteristic**	**Patients with:**		**Total**
		
	**Proven or Probable IPA**	**No IPA**	
**Sex:**			
Male	7	42	49
Female	6	26	32
**Age (years):**			
Median	60.91	50.40	53.68
Range	37.09 – 73.39	17.97 – 72.45	17.97 – 73.39
**Transplant type:**			
Allogeneic	6	40	46
Autologous	2	12	14
Non-Transplant	5	16	21
**Underlying disease:**			
ALL (Acute Lymphoblastic Leukemia)	0	8	8
AML (Acute Myeloid Leukemia)	4	12	16
AMM (Agnogenic Myeloid Metaplasia)	1	2	3
AMML (Acute Myelomonocytic Leukemia)	0	4	4
CLL (Chronic Lymphocytic Leukemia)	0	3	3
CML (Chronic Myeloid Leukemia)	0	7	7
HD (Hodgkin's Disease)	1	6	7
NHL (Non Hodgkin's Lymphoma)	1	8	9
MM (Multiple Myeloma)	3	4	7
RA (Refractory Anemia)	1	6	7
Other	2	8	10

### Analytical sensitivity and specificity of the qPCR assays

The *Aspergillus *qPCR standard curve of genomic *Aspergillus *DNA consistently yielded R^2 ^(goodness-of-fit) values > 0.98, which enabled quantification. The *Aspergillus *qPCR could reliably detect down to a threshold cycle (Ct) of 41 which is approximately equivalent to 1 fg of *Aspergillus *genomic DNA or a single copy of the target 18S rRNA gene.

To determine the specificity of the *Aspergillus *qPCR, 1 μg of human DNA and 1000 pg of fungal DNA from 29 species spanning 15 genera were tested in the *Aspergillus *qPCR assay. Only *Penicillium notatum *and *Paecilomyces variotii *had significant cross-reactivity, with similar levels of *Aspergillus *DNA reported with addition of 1000 pg DNA from these species. Cross-reactivity studies of the *Aspergillus *qPCR with human DNA revealed that 10 fg of *Aspergillus *DNA could be successfully amplified in the presence of 1 μg of human DNA per reaction. In actual BAL clinical samples, as little as 20 fg of *Aspergillus *DNA was detected in the presence of 550 ng of human DNA per reaction. These results demonstrate that very small quantities of *Aspergillus *DNA (< 1 genome) can be detected in a background of large amounts of human DNA (10^9 ^fold excess DNA by mass) using this assay. BAL samples from the first 48 episodes consisting of 10 true positives where an amplification product was detected were sequenced and confirmed to have DNA that matched the *Aspergillus *genus for each episode. Based on this high concordance rate sequencing was not performed for the subsequent 46 episodes.

The analytical sensitivity of the human 18S rRNA gene targeted extraction control qPCR was tested with human genomic DNA. The extraction control qPCR could reliably detect down to 37 Ct which is approximately equivalent to 1 pg of human genomic DNA or one-third of a human genome or 88 copies of the target 18S rRNA gene. The standard curve of human genomic DNA consistently yielded R^2 ^(goodness-of-fit) values > 0.98 for the human 18S rRNA gene qPCR, which enabled quantification of amounts of cellular material in BAL fluid.

### Extraction control qPCR

The extraction control qPCR qualitatively confirmed successful DNA extraction and estimated the amount of human genomic DNA present in all 144 BAL samples. The median amount of human genomic DNA per BAL pellet was 2.08 μg/pellet (52.1 ng per qPCR reaction) with a range of 9 ng to 58.8 μg per pellet. The amount of genomic DNA in the supernatant fraction was relatively low at a median of 88.8 ng/ml of BAL supernatant and had a range of 0.05 ng to 22.43 μg per ml BAL supernatant.

### Internal amplification control analysis of PCR inhibition

The IAC signal in the no-template controls was compared with the IAC signal of the BAL sample. A delay of 2 Ct (equivalent to a 3-fold change in quantity) or greater in duplicate qPCR reactions was used as a cut-off to classify a sample as having significant qPCR inhibition. The IAC multiplexed with the *Aspergillus *18S was more useful in detecting inhibition when compared with the IAC multiplexed with the extraction control assay. This is because the IAC signal in the extraction control was at a severe competitive disadvantage due to the large amounts of human DNA present in each sample. The IAC multiplexed with the *Aspergillus *18S assay detected significant inhibition in 11 samples. Inhibition in all these samples was overcome by re-extraction from the protein precipitation step onwards without significant loss of DNA as assessed by the extraction control qPCR (Fig. 1). When the re-extracted samples were assayed again, the IAC did not detect any inhibition.

### Contamination control

Sham digest controls were negative for *Aspergillus *DNA, showing that no fungal contamination was evident in the DNA extraction reagents. No-template controls were also negative, showing that fungal DNA contamination was not detected in the PCR reagents.

### Determination of the optimal fraction for detection of *Aspergillus *DNA in BAL fluid

After processing 66 BAL fluid samples from 48 episodes of pneumonia, data analysis was done to evaluate which fraction of BAL fluid contains the most *Aspergillus *DNA. Ten episodes were categorized as proven or probable IPA in this cohort of 48 episodes; within these 10 episodes, *Aspergillus *DNA was detected in 7 of 10 for the pellet fraction and in only 4 of 10 for the supernatant fraction. All positive supernatant fractions also had a positive pellet fraction. Of all qPCR positive BALs, an average of 98.3 ± 3.8% of total *Aspergillus *DNA from both fractions was seen in the pellet. Analysis of BAL pellet and supernatant results together conferred sensitivity and specificity identical to that of BAL pellet alone. It should be noted that although the supernatant fraction had low sensitivity (40%), it was highly specific in identifying episodes with proven or probable IPA (specificity 100%). Since BAL fluid supernatant did not appear to add meaningfully to the diagnostic yield, further analysis of BAL samples focused on analysis of BAL pellet fractions.

### Diagnostic utility of the *Aspergillus *qPCR, culture and histology

**Figure 2:**
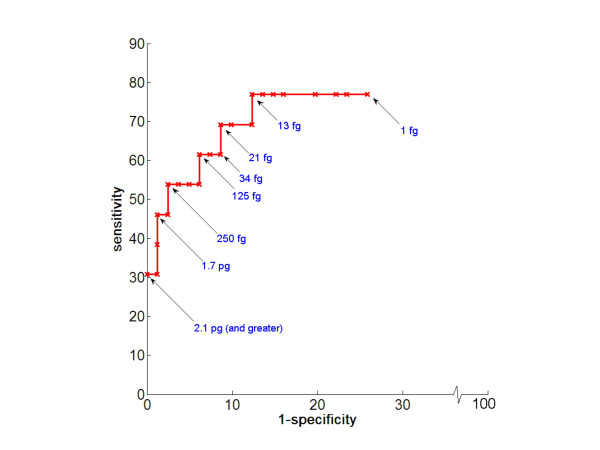
**Receiver-operating characteristic (ROC) analysis**. ROC curve depicting sensitivity versus 1-specificity of *Aspergillus *qPCR assay as a function of detection threshold of fungal burden in the BAL pellet (1 pg = 1000 fg). One genome of *A. fumigatus *corresponds to about 30-fg of genomic DNA and is estimated to have 28 copies of the target 18S rRNA gene.

Table 2 summarizes the key diagnostic parameters of the qPCR assay, culture, and histology in detecting the presence of *Aspergillus *in BAL fluid. ROC analysis of qPCR showed that a cut-off of 13 fg of *Aspergillus *genomic DNA per BAL pellet (corresponding to approximately 41 cycles) generated good sensitivity and specificity (Fig. 2). Based on this cut-off, the *Aspergillus *qPCR assay detected 10 of 13 episodes with proven or probable IPA (sensitivity 76.9%) and 8 out of 81 episodes without proven or probable IPA (specificity 90.1%). The positive and negative predictive values were 58% and 94%. For all BALs with any *Aspergillus *DNA detected by qPCR, the median quantity of *Aspergillus *DNA was 173 fg with a range of 4 fg to > 1500 pg per pellet.

**Table 2:** Summary of diagnostic performance in the detection of IPA.

**Diagnostic assay**	**BAL fraction**	**Sensitivity (%)**	**Specificity (%)**	**Positive Predictive Value (%)**	**Negative Predictive Value (%)**	**Positive Likelihood Ratio**	**Negative Likelihood Ratio**
qPCR, 13 fg	pellet	76.9 (50 – 92)	87.7 (79 – 93)	58	94	8.63 (3.2 – 11.9)	0.33 (0.1 – 0.7)
qPCR, 13 fg	supernatant	40 (17 – 69)	97.3 (86 – 99)	80.02	85.70	14.8 (1.9 – 118.1)	0.62 (0.37 – 1)
Culture	whole (unfractionated)	84.6 (58 – 96)	100 (95 – 100)	100	97.6	infinity	0.15 (0.04 – 0.6)
Histology	whole (unfractionated)	53.8 (29 – 77)	100 (95 – 100)	100	93.1	infinity	0.46 (0.3 – 0.8)
Culture or Histology	whole (unfractionated)	85.7 (60 – 96)	100 (95 – 100)	100	97.7	infinity	0.99 (0.04 – 0.5)

The range of values within brackets are estimated for a confidence interval of 95%. Thirteen femtograms (fg) of *Aspergillus *DNA was selected as our threshold for a positive PCR assay result, and is approximately equal to 1/3rd of an *Aspergillus fumigatus *genome. The supernatant fraction of the BAL was assayed for the first 48 episodes.

BAL culture was somewhat more sensitive than qPCR in detecting IPA (sensitivity 84.6 %) and had high specificity (100%). Histology on the other hand was less sensitive (53.8%), but had high specificity (100%). When culture and histology were used in combination, the sensitivity increased slightly to 85.7% and specificity remained at 100%. There was only a single episode with proven or probable IPA in which the histology was positive when the culture was negative. For this episode, the *Aspergillus *qPCR was convincingly positive with 2 pg of *Aspergillus *DNA found in the pellet. Two episodes with proven or probable IPA which were culture positive were not positive for qPCR or histology. It should be noted that in these episodes the culture was positive for *Aspergillus *at a single CFU level. One episode with proven or probable IPA showed no evidence of *Aspergillus *by culture or histology and was also negative by *Aspergillus *qPCR–this subject had a lung biopsy shortly after BAL fluid acquisition that confirmed IPA.

The Pearson correlation coefficient (r) calculated between the fungal burden estimated by qPCR and the number of colony forming units detected by culture in BAL was 0.93 (95% C.I. of 0.85 – 0.97, df = 25, p < 0.01), suggesting a strong relationship between these two independent measures of fungal burden. The 95% confidence intervals were estimated based on the Fisher r-to-z transformation.

### False positive (FP) and false negative (FN) results based on qPCR

Tables 3 and 4 show information about the false positive and false negative cases as identified by qPCR. Some patients did not meet standard criteria for IPA, but had consistently high levels of *Aspergillus *DNA in BAL fluid by qPCR based on repeated assays (Table 3). Since the no-template controls and digest controls were consistently negative, the FPs could not be directly attributed to contamination from DNA extraction or qPCR reagents.

**Table 3:** False positive cases.

**Pt.**	***Aspergillus *DNA (fg/pellet)**	**BAL Culture**	**Clinical Diagnosis**	**Computed tomography scan results and other clinical information**
1	5230	negative	DAH	Organizing pneumonia on lung biopsy with pulmonary hemorrhage; treated with ambisome empirically; No IPA at autopsy
2	230	negative	BOOP	Bilateral patchy opacities; no mould active antifungal therapy given
3	60	negative	Unknown	Nodular right middle lobe infiltrate treated with levofloxacin; exposure to hay
4	340	negative	IPA	Multiple bilateral nodules; treated as IPA with voriconazole + caspofungin
5	320	negative	DAH	Bilateral geographic grounds glass opacities; treated with caspofungin
6	80	negative	BOOP	Numerous bilateral ground glass opacities; treated with prednisone but no antifungal therapy
7	170	negative	Influenza pneumonia/PCP	Left lung infiltrates; no antifungal therapy except for Pneumocystis

Additional information about false positive (FP) cases as identified by qPCR. Pt.: patient; BOOP: Bronchiolitis obliterans with organizing pneumonia; DAH: Diffuse alveolar hemorrhage; PCP: Pneumocystis pneumonia.

**Table 4:** False negative cases.

**Pt.**	**BAL Culture**	**BAL Histology**	**Clinical Diagnosis**	**Human DNA (ng per reaction)**	**CT scan results and other clinical information**
8	negative	negative	IPA	0.23 from 1 BAL	No evidence of IPA in BAL; patchy bilateral infiltrates; lung biopsy 1 week later confirmed IPA by culture and histology
9	positive	negative	IPA DAH *Staphylococcus *pneumonia	33 and 72.5 from 2 BALs	1 CFU *A. fumigatus *in BAL fluid; patchy nodular infiltrates on CT; on ambisome for 10 days prior to bronchoscopy
10	positive	negative	IPA Legionella CMV pneumonia	10 to 218 from 4 BALs	1 CFU *A. niger *in BAL with CT scan showing halo sign, IPA confirmed at autopsy

Additional information about false negative (FN) cases as identified by qPCR. Pt.: patient; DAH: Diffuse alveolar hemorrhage; CMV: Cytomegalovirus.

All 3 FNs were negative for histology (Table 4). One (patient #9) was also negative for culture and the other two FNs (patient # 10 and 11) had positive culture values reported at a level of 1 CFU. These low or negative culture values could potentially reflect lower fungal burden in the BALs which could impact detection by qPCR. The IAC analysis ruled out PCR inhibition as a cause for FNs. In addition, the human genomic DNA amounts in the BAL pellets of FN samples were well within the tested limits of cross-reactivity with amplification by *Aspergillus *qPCR and hence inhibition due to human genomic DNA overload does not appear to be a factor affecting FN results. It is worthy to note that FN patient # 9 had the lowest amount of human genomic DNA per reaction of the entire study at 0.23 ng (Table 4). This could imply that enough cellular mass was not sampled during bronchoscopy which could in turn affect the chance of sampling fungal cells from the potential site of infection.

## Discussion

Despite the availability of new mould-active antifungal medications such as extended spectrum azoles (voriconazole, posaconazole) and echinocandins, aspergillosis remains a significant cause of death in patients with cancer. Delays in the institution of appropriate antifungal therapy may contribute to the high mortality seen with IPA, and the diagnosis of aspergillosis remains a clinical challenge, enhancing the potential for delay [1,3]. Molecular diagnostic techniques such as detection of *Aspergillus *DNA in BAL fluid using PCR are promising approaches that may facilitate rapid diagnosis, but published studies [12] tend to lack key quality control standards that are useful in identifying problems with false negative and false positive results within a study. Furthermore, the lack of appropriate controls affects the ability to coherently compare different published diagnostic PCR platforms for IPA [12,21,22,25]. We developed a qPCR approach for the diagnosis of IPA that incorporates rigorous quality control steps designed to determine if fungal contamination is introduced at the DNA extraction or PCR set up stages, if human DNA is present in the extracted samples and at what level (extraction control), if PCR inhibitors are present after DNA extraction and to what extent they cause inhibition (internal amplification control), and if large amounts of human genomic DNA impede the *Aspergillus *qPCR.

Many studies have been published describing various PCR assays for the diagnosis of IPA using BAL fluid, but the distribution of *Aspergillus *DNA in BAL fluid has not been systematically evaluated. We assayed the BAL pellet and supernatant fractions separately for the first 66 BALs (equivalent to 48 episodes) in order to identify the most useful fraction for diagnosis. Our results showed that 98.3% of *Aspergillus *DNA in BAL fluid is cell-associated, most likely as either intact fungal cells or as fungi engulfed by leukocytes. The most significant implication of this result is that the diagnostic yield may increase by centrifuging large volumes of BAL fluid and subjecting the pellet to a single extraction. There was no diagnostic benefit from analyzing BAL supernatant as it was positive only when the pellet was positive for *Aspergillus *DNA. The *Aspergillus *DNA detected in the supernatant may have resulted from low-level lysis of *Aspergillus *cells and release of DNA either in vivo (bronchial lining fluid), or in vitro after BAL fluid collection. Attempts to concentrate *Aspergillus *DNA in the supernatant fraction using ultrafiltration provided no additional value but rather resulted in losses of DNA when compared to the original supernatant extract (data not shown). The BAL fluid pellet appears to be the best fraction for use in the diagnosis of IPA.

*Aspergillus *conidia are ubiquitous in the environment, creating the potential for false positive fungal PCR results when highly sensitive PCR assays are employed [21]. Fungal cells or fungal DNA can enter the assay process at numerous points, including at the time of BAL collection, during DNA extraction, or at qPCR set up. UV irradiation and ultrafiltration have been previously used to control PCR contamination [26-28]. Apart from processing samples in a laminar flow hood within a laboratory that was exclusively used for pre-PCR processing, we used UV irradiation, filtration of solutions, and baking of beads and glassware as additional tools to eliminate potential contaminants present in the extraction and PCR reagents. The Yeast Cell Lysis Solution™ and the Protein Precipitation Reagent™ (Epicentre^® ^Biotechnologies, Madison, WI) used in DNA extraction could be UV irradiated without loss of function (data not shown). The silicon carbide sharps used in the bead beating step of DNA extraction were specifically chosen from a wide array of materials for their ability to remain chemically and physically stable through a 2-day baking period required to eliminate any contaminating nucleic acids. The organic solvents used in DNA extraction were filtered through a membrane with MWCO of 30 kDa. The qPCR mastermix reagents were carefully selected such that they could all be filtered through a membrane of 100 kDa MWCO. The nucleotide cut-off for a 30 kDa filter was 60 bases of single stranded DNA and 50 bp double stranded DNA, and for a 100 kDa filter was 300 bases single stranded DNA and 125 bp double stranded DNA. Even though our *Aspergillus *qPCR amplicon was 114 bp long (estimated MW of 70 kDa in its double stranded form), we could consistently prevent contamination of the PCR reagents. To minimize any contamination emerging from the IAC qPCR, the IAC primers, probe and template, which were multiplexed with the target qPCR assays, were also filtered as part of the mastermix. The size of the IAC template was designed to be 105 bases long such that it could easily filter through a membrane of 100 kDa MWCO. Another source of contamination in PCR assays may arise from amplicon carry-over contamination from previous PCR runs of the same assay. In addition to strictly isolating pre- from post-PCR work, a uracil-N-glycosylase (UNG) enzyme step was incorporated prior to PCR in combination with use of the nucleotide 2'-deoxyuridine 5'-triphosphate (dUTP) to degrade previous PCR products and prevent carry-over contamination. The water-only sham digest controls and no-template PCR controls used with every experiment were consistently negative confirming that we were able to control contamination originating from the DNA extraction and PCR set ups.

Another factor contributing to FPs can be cross-reactivity of the *Aspergillus *qPCR assay with non-*Aspergillus *fungi. Extensive analytical specificity testing showed that among the 23 non-*Aspergillus *fungal species, our *Aspergillus *qPCR assay had significant cross-reactivity only with *Penicillium chrysogenum *and *Paecilomyces variotii*. *P. chrysogenum *is a ubiquitous fungus closely related to *A. fumigatus*. It is rarely associated with human opportunistic infections. *P. variotii *is an opportunistic human pathogen, but voriconazole, which is considered first line therapy targeting *Aspergillus *species, is also active against *P. variotii*. Thus the clinical ramifications of incorrectly calling a *Paecilomyces *infection an *Aspergillus *infection are likely to be small. The galactomannan antigen assay for diagnosis of aspergillosis is also susceptible to false positive results due to cross-reactivity with antigens from these two fungal species [10].

Although qPCR assays can detect down to a few target molecules of template per reaction, DNA extraction of fungal pathogens from clinical samples remains the bottleneck of PCR diagnostics [29-31]. Each BAL sample may consist of sterile saline (lavage fluid), fungal cells, biological components which may be PCR inhibitors (e.g. heme and mucus), and a large amount of human cells. Our previous work evaluated the utility of 6 commercial fungal DNA extraction kits [30]. Based on optimizing fungal DNA yields and minimizing FP and FN results, we selected the MasterPure™ Yeast DNA Purification Kit (Epicentre^® ^Biotechnologies, Madison, WI) for this study. The DNA extraction protocol was further optimized by adding silicon carbide sharps for lysis of fungal cell wall which significantly enhanced extraction yields. The extraction control qPCR qualitatively confirmed successful extractions and gave a quantitative measure of the amount of human genomic DNA present in every BAL extract. In addition, it helped guarantee that the BAL fluid contacted a human mucosal surface and that DNA was not significantly degraded. Although processing fungal cells in parallel to asses extraction efficiency has been suggested as a more accurate measure, such an approach brings with it the high probability of introducing fungal contamination in the pre-PCR processing lab and is thus a serious drawback. We avoided processing and extraction of fungal cells from external sources to minimize the potential for false positive PCR results. We verified the ability of our *Aspergillus *qPCR assay to successfully amplify 10 fg of *A. fumigatus *genomic DNA (< 1 *Aspergillus *genome) in the presence of 1 μg of human genomic DNA per reaction (10^9 ^fold excess human DNA). The human 18S rRNA gene PCR extraction control measurements helped validate that human genomic DNA in actual PCR reactions derived from BAL fluid was well within these limits, providing evidence that human DNA did not interfere with assay performance leading to false negative results.

It has been suggested that an IAC is critical for assessing PCR inhibition in every sample to rule out inhibition as a cause for FNs [20,32]. Very few studies related to fungal PCR diagnostics analyzing BAL fluid have incorporated an IAC [33,34], only one of which focused on *Aspergillus *detection in BAL. Our IAC was a truncated version of an exogenous DNA template derived from the jellyfish aequorin gene previously used in PCR studies for the diagnosis of cytomegalovirus disease [24]. Known amounts of IAC template introduced in the multiplexed qPCR mastermix enabled reliable quantification of inhibition in the *Aspergillus *or extraction control qPCRs. Because the IAC was added during the qPCR stage, it was unaffected by other variables of the process (like DNA extraction) and therefore it exclusively monitored inhibition in qPCR. In addition, the multiplexed IAC amplified with primers and probe independent from the target and its reaction kinetics were optimized such that it did not affect the analytical sensitivity of the target qPCR assay (as confirmed by the positive *A. fumigatus *standards in each experiment). The IAC qPCR detected inhibition in 7.6% (11 out of 144) of the BAL samples. Re-extraction of DNA eliminated PCR inhibition in all samples without significant losses of DNA. Therefore, the IAC ruled out qPCR inhibition as a cause for FNs in this study. It is also noteworthy to mention that the IAC qPCR multiplexed with the *Aspergillus *qPCR assay did not manifest any inhibition even in the presence of human genomic DNA as high as 1.5 μg per reaction. This implies that the IAC was monitoring for qPCR inhibition independent of the large quantities of human genomic DNA found in extracted BAL fluid.

A ROC curve of the *Aspergillus *qPCR assay depicted diagnostic sensitivity versus 1-specificity as a function of detection threshold of fungal burden (e.g. femtograms of DNA). This was useful in identifying the threshold of detection with an optimal trade-off between diagnostic sensitivity and specificity. The ROC analysis showed that a detection threshold of 13 fg of *Aspergillus *DNA per pellet generated a sensitivity and specificity of 76.9% and 87.7%, respectively (Fig. 2). These data compare favorably to the sensitivity and specificity of both PCR and galactomannan antigen studies using BAL fluid published previously [10,12]. At this detection threshold and a prevalence of proven or probable IPA of 13.8%, the NPV of *Aspergillus *qPCR assay was high at 94% and the PPV was 58%, consistent with results from other studies using BAL to diagnose IPA [16]. In addition, the PPV of our qPCR was similar to the FDA approved serum GM test used on hematopoietic stem cell transplant patients [35,36]. The lower PPV reflects the relatively low prevalence of IPA when analyzed on a per episode basis. A high NPV is useful as it suggests that a patient is unlikely to have IPA if the test is negative, and this may spare the patient from receiving unnecessary antifungal treatment that was started empirically, though these results do not rule out infection with another fungus.

Some patients (7) did not meet standard criteria for IPA, but had consistently high levels of *Aspergillus *DNA in BAL fluid by qPCR, as documented by repeated detection in multiple qPCR assays performed on different days (Table 3). These cases may be false positives due to fungal colonization of the airways, fungal contamination at the time of BAL collection, or true positives indicative of shortcomings in the EORTC/MSG criteria for defining IPA. Several of these patients had a diagnosis of idiopathic pneumonia syndrome or related pulmonary conditions after hematopoietic cell transplantation and were treated with mould-active antifungal medications empirically, thus the diagnosis of IPA cannot be completely excluded despite the absence of formal criteria for IPA. On the other hand, other patients did not receive mould-active antifungal therapy and did not appear to develop sequelae of IPA despite absence of treatment, suggesting that these episodes are definite false positives.

Among the 3 patients with false negative results, one had no evidence of *Aspergillus *in the original BAL sample using any diagnostic method, but proved to have IPA based on a subsequent lung biopsy. This false negative sample had the lowest amount of cellular material of all the BALs processed in this study which may reflect inadequate sampling of the lung segment at the time of bronchoscopy with BAL. Two patients with false negative PCRs for *Aspergillus *had 1 CFU of *Aspergillus *detected in BAL fluid by culture. This failure to detect *Aspergillus *DNA in the BAL fluid from these 2 subjects may reflect the low burden of fungal organisms, or may reflect the fact that these culture results are false positives (laboratory contamination), leading to improper classification using EORTC/MSG criteria.

There are several limitations of our study. First, the BAL procedure is highly variable which may impact the amount of fungal cells sampled from the site of infection. Second, significant portions of BAL were sent for other microbiological tests like histology and culture. There could be an impact of uneven distribution of fungi in the BAL between aliquots submitted for these various tests. In addition, the sensitivity of our study may have increased if larger volumes of BAL were processed. Third, we do not have serum or BAL galactomannan data for these subjects, which limit our ability to compare PCR performance with another molecular diagnostic test. Fourth, based on the EORTC/MSG criteria, culture was primarily used as a gold standard in defining IPA. This may have led to an overestimate of sensitivity and specificity of culture in our study.

In our study, quantitative PCR was about as sensitive as culture or culture with histology combined (Table 2). In contrast to culture, qPCR results can be generated in one day. Although culture was equally sensitive in detecting IPA in this study, it should be noted that this result probably reflects the critical role that cultivation played in defining subjects with IPA using EORTC/MSG criteria in this study. Several studies in the past have reported the sensitivity of BAL culture to be relatively low (< 50%) [4,7,8]. Of the 13 episodes with proven or probable IPA, culture and *Aspergillus *qPCR (with a 13 fg detection threshold) were concurrently positive for 8 episodes. There were 2 episodes when culture was negative and qPCR was positive and 3 episodes when culture was positive and qPCR negative. In addition, qPCR was always positive when histology was positive. This implies that when qPCR is conjunctively used with culture, sensitivity of detecting IPA could approach 100%, though much larger numbers of samples will be needed to define the true sensitivity and specificity of the qPCR assay. The significant correlation of fungal burden estimated by qPCR and the CFUs reported by culture tests implies qPCR is highly likely to be positive when culture and/or histology are positive. This result is in concordance with several published studies [15,18,34,37].

## Conclusion

Our *Aspergillus *qPCR assay detected *Aspergillus *DNA in 76.9% of subjects with proven or probable IPA when the concentrated BAL fluid pellet fraction was used for diagnosis. There was no benefit from analyzing the BAL supernatant fraction. Use of both extraction and amplification controls provided optimal quality control for interpreting qPCR results. PCR inhibitors detected in samples by the IAC could be removed with re-extraction of the DNA. Some patients did not meet standard criteria for IPA, but had consistently high levels of *Aspergillus *DNA in BAL fluid by qPCR suggesting that the FPs may result from colonization of the airways or shortcomings of the MSG/EORTC classification criteria. Future studies will involve additional testing of BAL samples where the performance of galactomannan antigen assay is also evaluated. The rigorous quality control steps incorporated in our qPCR assay significantly enhance the reliability of the results and therefore may increase our understanding of the true potential of qPCR for the diagnosis of IPA.

## Competing interests

The authors declare that they have no competing interests.

## Authors' contributions

PDK helped to design the study, developed and optimized the qPCR assays, performed data analysis, and drafted the manuscript. DLK participated in the design of the study, assisted with the development, optimization, and implementation of the qPCR assays, and helped to draft the manuscript. RCH facilitated the collection and storage of BAL samples, was responsible for histological analysis of samples, and critically reviewed the manuscript. DNF conceived of the study, participated in the design of the study, categorized patients into diagnostic groups, assisted with data analysis, and helped to draft and review the manuscript. All authors read and approved the final manuscript.

## Pre-publication history

The pre-publication history for this paper can be accessed here:



## References

[B1] Segal BH, Walsh TJ (2006). Current approaches to diagnosis and treatment of invasive aspergillosis. Am J Respir Crit Care Med.

[B2] McNeil MM, Nash SL, Hajjeh RA, Phelan MA, Conn LA, Plikaytis BD, Warnock DW (2001). Trends in Mortality Due to Invasive Mycotic Diseases in the United States, 1980–1997.. Clinical Infectious Diseases.

[B3] Munoz P, Guinea J, Bouza E (2006). Update on invasive pulmonary aspergillosis: clinical and diagnostic aspects. Clin Microbiol Infect.

[B4] Reichenberger F, Habicht JM, Gratwohl A, Tamm M (2002). Diagnosis and treatment of invasive pulmonary aspergillosis in neutropenic patients. Eur Respir J.

[B5] Latge JP (1999). Aspergillus fumigatus and aspergillosis. Clin Microbiol Rev.

[B6] Reichenberger F, Habicht J, Matt P, Frei R, Soler M, Bolliger CT, Dalquen P, Gratwohl A, Tamm M (1999). Diagnostic yield of bronchoscopy in histologically proven invasive pulmonary aspergillosis. Bone Marrow Transplant.

[B7] Levy H, Horak DA, Tegtmeier BR, Yokota SB, Forman SJ (1992). The value of bronchoalveolar lavage and bronchial washings in the diagnosis of invasive pulmonary aspergillosis. Respir Med.

[B8] Saito H, Anaissie EJ, Morice RC, Dekmezian R, Bodey GP (1988). Bronchoalveolar lavage in the diagnosis of pulmonary infiltrates in patients with acute leukemia. Chest.

[B9] Adam O, Auperin A, Wilquin F, Bourhis JH, Gachot B, Chachaty E (2004). Treatment with piperacillin-tazobactam and false-positive Aspergillus galactomannan antigen test results for patients with hematological malignancies. Clin Infect Dis.

[B10] Aquino VR, Goldani LZ, Pasqualotto AC (2007). Update on the contribution of galactomannan for the diagnosis of invasive aspergillosis. Mycopathologia.

[B11] Kedzierska A, Kochan P, Pietrzyk A, Kedzierska J (2007). Current status of fungal cell wall components in the immunodiagnostics of invasive fungal infections in humans: galactomannan, mannan and (1-->3)-beta-D-glucan antigens. Eur J Clin Microbiol Infect Dis.

[B12] Tuon FF (2007). A systematic literature review on the diagnosis of invasive aspergillosis using polymerase chain reaction (PCR) from bronchoalveolar lavage clinical samples. Rev Iberoam Micol.

[B13] Buchheidt D, Baust C, Skladny H, Ritter J, Suedhoff T, Baldus M, Seifarth W, Leib-Moesch C, Hehlmann R (2001). Detection of Aspergillus species in blood and bronchoalveolar lavage samples from immunocompromised patients by means of 2-step polymerase chain reaction: clinical results. Clin Infect Dis.

[B14] Hayette MP, Vaira D, Susin F, Boland P, Christiaens G, Melin P, De Mol P (2001). Detection of Aspergillus species DNA by PCR in bronchoalveolar lavage fluid. J Clin Microbiol.

[B15] Musher B, Fredricks D, Leisenring W, Balajee SA, Smith C, Marr KA (2004). Aspergillus galactomannan enzyme immunoassay and quantitative PCR for diagnosis of invasive aspergillosis with bronchoalveolar lavage fluid. J Clin Microbiol.

[B16] Raad I, Hanna H, Huaringa A, Sumoza D, Hachem R, Albitar M (2002). Diagnosis of invasive pulmonary aspergillosis using polymerase chain reaction-based detection of aspergillus in BAL. Chest.

[B17] Rantakokko-Jalava K, Laaksonen S, Issakainen J, Vauras J, Nikoskelainen J, Viljanen MK, Salonen J (2003). Semiquantitative detection by real-time PCR of Aspergillus fumigatus in bronchoalveolar lavage fluids and tissue biopsy specimens from patients with invasive aspergillosis. J Clin Microbiol.

[B18] Sanguinetti M, Posteraro B, Pagano L, Pagliari G, Fianchi L, Mele L, La Sorda M, Franco A, Fadda G (2003). Comparison of real-time PCR, conventional PCR, and galactomannan antigen detection by enzyme-linked immunosorbent assay using bronchoalveolar lavage fluid samples from hematology patients for diagnosis of invasive pulmonary aspergillosis. J Clin Microbiol.

[B19] Spiess B, Buchheidt D, Baust C, Skladny H, Seifarth W, Zeilfelder U, Leib-Mosch C, Morz H, Hehlmann R (2003). Development of a LightCycler PCR assay for detection and quantification of Aspergillus fumigatus DNA in clinical samples from neutropenic patients. J Clin Microbiol.

[B20] Paterson RR (2007). Internal amplification controls have not been employed in fungal PCR hence potential false negative results. J Appl Microbiol.

[B21] Loeffler J, Hebart H, Bialek R, Hagmeyer L, Schmidt D, Serey FP, Hartmann M, Eucker J, Einsele H (1999). Contaminations occurring in fungal PCR assays. J Clin Microbiol.

[B22] Bretagne S (2003). Molecular diagnostics in clinical parasitology and mycology: limits of the current polymerase chain reaction (PCR) assays and interest of the real-time PCR assays. Clin Microbiol Infect.

[B23] Ascioglu S, Rex JH, de Pauw B, Bennett JE, Bille J, Crokaert F, Denning DW, Donnelly JP, Edwards JE, Erjavec Z, Fiere D, Lortholary O, Maertens J, Meis JF, Patterson TF, Ritter J, Selleslag D, Shah PM, Stevens DA, Walsh TJ (2002). Defining opportunistic invasive fungal infections in immunocompromised patients with cancer and hematopoietic stem cell transplants: an international consensus. Clin Infect Dis.

[B24] Limaye AP, Huang ML, Leisenring W, Stensland L, Corey L, Boeckh M (2001). Cytomegalovirus (CMV) DNA load in plasma for the diagnosis of CMV disease before engraftment in hematopoietic stem-cell transplant recipients. J Infect Dis.

[B25] Ferns RB (2006). Evaluation of the role of real-time PCR in the diagnosis of invasive aspergillosis. Leuk Lymphoma.

[B26] Ou CY, Moore JL, Schochetman G (1991). Use of UV irradiation to reduce false positivity in polymerase chain reaction. Biotechniques.

[B27] Wages JM, Cai D, Fowler AK (1994). Removal of contaminating DNA from PCR reagents by ultrafiltration. Biotechniques.

[B28] Jordan JA, Durso MB (2000). Comparison of 16S rRNA gene PCR and BACTEC 9240 for detection of neonatal bacteremia. J Clin Microbiol.

[B29] Loffler J, Hebart H, Schumacher U, Reitze H, Einsele H (1997). Comparison of different methods for extraction of DNA of fungal pathogens from cultures and blood. J Clin Microbiol.

[B30] Fredricks DN, Smith C, Meier A (2005). Comparison of six DNA extraction methods for recovery of fungal DNA as assessed by quantitative PCR. J Clin Microbiol.

[B31] Griffiths LJ, Anyim M, Doffman SR, Wilks M, Millar MR, Agrawal SG (2006). Comparison of DNA extraction methods for Aspergillus fumigatus using real-time PCR. J Med Microbiol.

[B32] Hoorfar J, Malorny B, Abdulmawjood A, Cook N, Wagner M, Fach P (2004). Practical considerations in design of internal amplification controls for diagnostic PCR assays. J Clin Microbiol.

[B33] Caliendo AM, Hewitt PL, Allega JM, Keen A, Ruoff KL, Ferraro MJ (1998). Performance of a PCR assay for detection of Pneumocystis carinii from respiratory specimens. J Clin Microbiol.

[B34] Bretagne S, Costa JM, Marmorat-Khuong A, Poron F, Cordonnier C, Vidaud M, Fleury-Feith J (1995). Detection of Aspergillus species DNA in bronchoalveolar lavage samples by competitive PCR. J Clin Microbiol.

[B35] Herbrecht R, Letscher-Bru V, Oprea C, Lioure B, Waller J, Campos F, Villard O, Liu KL, Natarajan-Ame S, Lutz P, Dufour P, Bergerat JP, Candolfi E (2002). Aspergillus galactomannan detection in the diagnosis of invasive aspergillosis in cancer patients. J Clin Oncol.

[B36] Pfeiffer CD, Fine JP, Safdar N (2006). Diagnosis of invasive aspergillosis using a galactomannan assay: a meta-analysis. Clin Infect Dis.

[B37] Spreadbury C, Holden D, Aufauvre-Brown A, Bainbridge B, Cohen J (1993). Detection of Aspergillus fumigatus by polymerase chain reaction. J Clin Microbiol.

